# Chemical Composition of the Essential Oils of the Iberian Peninsula Endemic Species *Eryngium dilatatum* Lam.

**DOI:** 10.3390/molecules29030562

**Published:** 2024-01-23

**Authors:** Jesús Palá-Paúl, María José Pérez-Alonso, Ana C. Soria, Joseph J. Brophy

**Affiliations:** 1 Departamento de Biodiversidad, Ecología y Evolución, Facultad de Ciencias Biológicas, Universidad Complutense de Madrid, 28040 Madrid, Spain; 2 Instituto de Química Orgánica General (IQOG-CSIC), Juan de la Cierva 3, 28006 Madrid, Spain; acsoria@iqog.csic.es; 3 School of Chemistry, The University of New South Wales, Sydney, NSW 2052, Australia; j.brophy@unsw.edu.au

**Keywords:** *Eryngium dilatatum*, germacrene D, octanal, spathulenol, essential oil, chemical composition

## Abstract

*Eryngium dilatatum* Lam. is a thorny Iberian Peninsula endemic species belonging to the Apiaceae family that has not been previously analysed from a chemical point of view. Following our studies on this genus, we characterized the chemical composition of the essential oils from the different parts (inflorescences, stems + leaves, and roots) of this species; these parts were gathered in Cádiz (Spain). The specimens were collected in July during the flowering period and air-dried before the oil extraction by hydro-distillation. The essential oils were analysed by gas chromatography and gas chromatography coupled with mass spectrometry. The different parts of the plant yielded low amounts of pale yellow oil, with the roots being the fraction that provided the lowest amount of oil. The chemical characterization of the essential oils showed qualitative and quantitative differences between the fractions examined, but all of them showed the same principal compound, germacrene D (9.1–46.5%). Similarly, all the fractions shared most of their representative constituents, with their percentage compositions being different from one sample to the other: α-cadinol (3.8%), bicyclogermacrene (3.5%), octanal (3.1%), and spathulenol (2.5%) were found in the inflorescences; octanal (8.1%), α-cadinol (3.7%), δ-cadinene (3.6%), (*E*)-caryophyllene (2.6%), bicyclogermacrene (2.5%), and spathulenol (2.4%) were found in the stems and leaves; and spathulenol (4.6%), α-cadinol (4.4%), khusinol (3.2%), α-muurolol (3.1%), and δ-cadinene (2.6%) were found in the roots. As far as we know, this is the first report about the chemical composition of this endemic species of the Iberian Peninsula. It contributes to the knowledge of this species and to the genus to which it belongs. This species could be considered as a natural source of germacrene D, which is a sesquiterpene hydrocarbon with active properties.

## 1. Introduction

**Figure 1:**
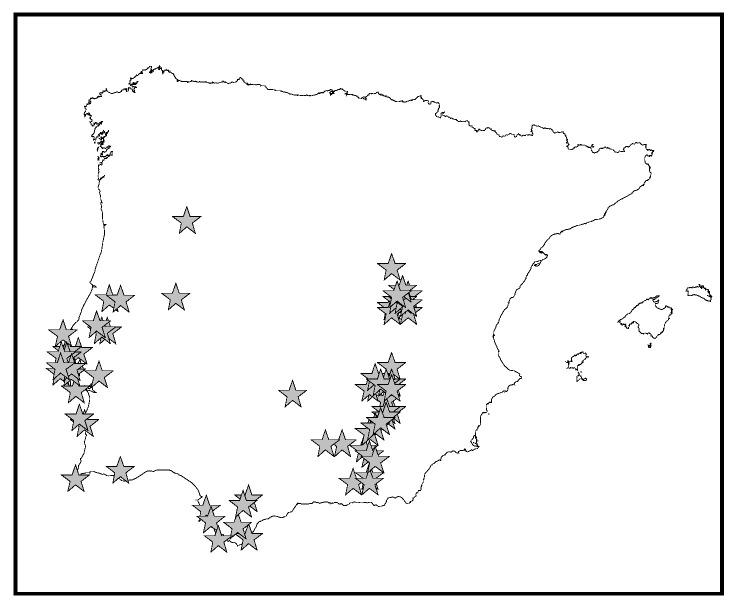
Distribution of *Eryngium dilatatum* Lam. in the Iberian Peninsula.

The genus *Eryngium* L. belongs to the Apiaceae family and, with about 250 species, has a cosmopolitan distribution. This genus includes annual and perennial herbs with hairless and spiny leaves, which present dome-shaped umbels of steely blue or white flowers with whorls of spiny basal bracts. Only fourteen of the twenty-six species described in Flora Europaea grow wildly in the Iberian Peninsula [1]. *Eryngium dilatatum* Lam. is a perennial herb (from 5 to 40 cm) with erect stems that are rarely branched; their basal leaves, which are slightly coriaceous and obovate, are persistent. From June to August, it presents bluish inflorescences in pedunculated capitula from 0.5 to 1.5 cm. *E. dilatatum* is one of the four *Eryngium* species endemic to the Iberian Peninsula. It grows in dry places in Portugal and Spain with a disjunct distribution (Figure 1).

Despite the high number of species belonging to this genus, only a few of them have previously been investigated. With regard to the species distributed in the Iberian Peninsula, this could be caused by the small size of some species, such as *E. tenue* Desf. or *E. galioides* Lam., their spiny leaves that make their recollection and study difficult, or the inaccessible environment where they grow, as in the case of *E. glaciale* Boiss. or *E. bourgatii* Gouan. In general, most of the studies related to this genus could be grouped according to their chemistry (flavonoids, phenolic contents, phytochemistry, etc.), genetic diversity, and biological properties (analgesic, antibacterial, anticlastogenic, anti-inflammatory, antimicrobial, antioxidant, cytotoxic activity, larvicidal, long-term memory, molluscicidal, parasiticidal, etc.) [2,3,4,5,6,7,8,9,10,11,12,13,14,15,16,17,18,19,20,21,22,23,24,25,26,27,28,29,30,31,32,33,34]. The essential oils of the *Eryngium* genus have not been widely investigated; the majority of the reports addressing their compositions have been published in the last 20 years [35,36,37,38,39,40,41,42,43,44,45,46,47,48,49,50,51,52,53,54,55,56,57,58,59,60,61,62,63,64,65,66,67,68,69,70,71,72,73,74,75,76,77,78,79,80,81,82,83,84,85,86].

The main compounds and the yield, as given by the data that are available, of the essential oils extracted from the species which grow in the Iberian Peninsula are shown in Table 1. It is worth noting that only seven of the fourteen species present in this area have been analysed. According to these results, the *Eryngum* genus shows low amounts of essential oils with yields varying between 0.1 and 0.82%. Normally, the fraction with the highest amount of oil is the one corresponding to the inflorescences, which could be related to the pollination of the species. However, the species studied to date showed different chemical compositions according to their ecology. We found that the species that grow at high altitudes under hard climatic conditions contain a large amount of diterpenes; these are the compounds that make up the predominant fraction of the oil in *Eryngium bourgatii* Gouan [73] and *E. glaciale* Boiss. [74]. In fact, the altitude seems to affect the chemical composition of the Iberian endemic species *E. duriaei* J. Gay ex Boiss. The populations of this species that grow below 1700 m showed α-neocallitropsene (28–53%), β-betulenal (8.5–15.8%), and 14-hydroxy-β-caryophyllene (5.8–13.7%) as the main compounds, while caryophyllene oxide (47%) and *E*-caryophyllene (6%) were identified in the analysed population growing above this altitude [56]. *E. maritimum* L. [81] is a species that grows in sandy places on the coast and contains sesquiterpenes as its principal fraction, while monoterpenes are the largest fraction in the oils of *E. corniculatum* Lam. [71], which grows on the margins of seasonal lakes. The studies on the chemical composition of *E. campestre* growing under different soil types have also been reported [69].

**Table 1:** Main constituents of the essential oils of the *Eryngium* species growing wild in the Iberian Peninsula.

Species	Main Components	Yield (%)	Ref.
*E. aquifolium* Cav.	Inflorescence oil: germacrene D (30.3%) and sesquicineole (26.7%)	0.81	[65]
	Stem and leaf oil: germacrene D (46.0%) and myrcene (13.8%)	0.41	
	Root oil: phyllocladene isomer (63.6%)	0.18	
*E. bourgatii* Gouan	Inflorescence oil: phyllocladene (37.6%) and bicyclogermacrene (15.1%)	0.33	[73]
	Stem and leaf oil: phyllocladene (20.4%), γ-muurolene (11.8%) and (*E*)-caryophyllene (10.1%)	0.11	
	Root oil: γ-muurolene (15.4%) and phyllocladene (15.0%)	0.20	
*E. campestre* L.	Inflorescence oil: germacrene D (30.3–40.3%), β-curcumene (0.7–22.2%), myrcene (3.0–21.7%), (E)-β-farnesene (0.1–19.0%).	0.1–0.4	[69]
	Stem and leaf oil: germacrene D (31.1–42.4%), myrcene (0.5–23.15)	0.1–0.2	
*E. corniculatum* Lam.	Inflorescence oil: 2,4,6-trimethylbenzaldehyde (50.8%), α-pinene (4.0%)	0.82	[72]
	Stem and leaf oil: 2,4,6-trimethylbenzaldehyde (50.0%), 2,4,5-trimethylbenzaldehyde (3.8%)	0.49	
	Root oil: 2,4,6-trimethylbenzaldehyde (29.8%), phyllocladene isomer (13.0%), (*E*)-nerolidol (9.4%)	0.22	
*E. duriaei* J. Gay ex Boiss.	Populations below 1700 m: α-neocallitropsene (28–53%), β-betulenal (8.5–15.8%) and 14-hydroxy-β-caryophyllene (5.8–13.7%)	0.2–0.3	[56]
	Population over 1700 m: caryophyllene oxide (47%) and *E*-caryophyllene (6%)		
*E. glaciale* Boiss.	Inflorescence oil: phyllocladene isomer (43.5%), (*E*)-caryophyllene (15.2%) and valencene (11.5%)	0.16	[74]
	Stem and leaf oil: phyllocladene isomer (41.3%)	0.26	
	Root oil: phyllocladene isomer (49.4%) and linalool (19.1%).	0.30	
*E. maritimum* L.	Aerial parts: germacrene D (43.1–42.4%) and 9-muurolen-15-aldehyde (22.4–16.4%)	--	[81]
	Roots: γ-guaiene (40.2%), 2,3,4-trimethylbenzaldehyde (24.5%) and germacrene D (10.6%)	--	

Nowadays, there is a great concern about biodiversity conservation and the modification of habitats caused by the climatic change, particularly in areas that are susceptible to extended droughts, such as in Spain. One of the best ways to contribute to this conservation is to provide as much knowledge as possible on the ecological valence of species growing wild in these areas. Following our work with other *Eryngium* species, the aim of this particular study was to analyse the essential oils of the different parts of *E. dilatatum* and to compare their chemical composition with those of the other Iberian species studied to date. This study contributes to the deepening of the knowledge on the chemical composition of the species of the *Eryngium* genus. Deepening the knowledge on the variability and wealth of compounds that this genus presents is essential to the understanding of its diversity and adaptation to the different environments where it grows. As far as we know, this is the first report on the volatile components of this species which is endemic to the Iberian Peninsula.

## 2. Results and Discussion

The different fractions, voucher numbers, locality, and oil yield of the population of *E. dilatatum* considered in this work are presented in Table 2. According to these results, the aerial fractions showed a similar yield (0.29–0.33%), whereas the roots produced a low amount of oil (0.14%). These results are in agreement with those previously reported for other Iberian species of this genus (Table 1). Usually, the roots are the part of the plant with lowest amount of oil. Most of the *Eryngium* species are perennial or geophyte plants; so, they can synthesize or accumulate oil in this part of the plant during the winter. In fact, perennial species growing at a high altitude seem to accumulate oils in this part of the plant (*E. bourgatii* and *E. glaciale*). As has previously been proposed for this genus [13,56,69,71,77], the type of soil, the climatic conditions, and the water availability could affect the production of essential oil. The species that grow in dry places contain low amounts of oils (0.1–0.33%), while the species that grow close to lakes (e.g., *E. corniculatum*) show higher amounts of oil (0.22–0.82%) [72]. The available water seems to affect the metabolic route of the terpenoid compounds in this genus, proving the adaptation of the species to the environment where they grow. However, this hypothesis should be confirmed under controlled experimental conditions or by in vitro cultures of each species. Another species of this genus growing in a similar habitat, *E. aquifolium* [65], showed a yield that was two times higher in the inflorescences, which could contribute to the pollination success. It would be interesting to study the phenology of the essential oils of *E. dilatatum* to determine their variation during the year and the possible effect of the flowering and fruiting period. Additionally, those phenology studies could also be carried out on different populations to evaluate whether the yield and chemical composition of the essential oils of this species vary according to ecological factors, as has previously been reported for other species of this genus [56,69]. However, the present study was a first contribution to the chemical composition of this species; therefore, it was limited to a single population.

The components identified from the different parts of *Eryngium dilatatum*, their retention indices, and their percent composition are summarised in Table 3, where all the compounds are arranged in order of their elution on the DB-1 column. The retention indices of these compounds on a DB-Wax column are also listed in brackets in this table. A total of 79 compounds were identified, representing 76.2–97.3% of the total oil obtained from the different parts of the plant studied. As we mentioned above, the root fraction was the source of the lowest amount of oil but, with regard to the chemical composition, few qualitative or quantitative differences in the oil content of the three fractions analysed were observed. Although all the fractions analysed showed the same principal compound, germacrene D (9.1–46.5%), and shared most of their representative constituents, their percentage compositions differed with the plant part considered: α-cadinol (3.8%), bicyclogermacrene (3.5%), octanal (3.1%), spathulenol (2.5%), and α-muurolol (2.5%) were found in the inflorescences; octanal (8.1%), α-cadinol (3.7%), δ-cadinene (3.6%), (*E*)-caryophyllene (2.6%), bicyclogermacrene (2.5%), and spathulenol (2.4%) were found in the stems and leaves; and spathulenol (4.6%), α-cadinol (4.4%), khusinol (3.2%), α-muurolol (3.1%), δ-cadinene (2.6%), 14-oxy-α-muurolene (2.6%), and carotol (2.5%) were found in the roots. Five compounds could not be identified, and their mass spectral fragmentation patterns are provided at the end of Table 3.

The same main compound was identified in all the analysed fractions. Other species of this genus also shared their major constituents, although each species seems to have plasticity in the chemical composition. To date, the studied species growing in the Iberian Peninsula show two patterns, *E. bourgatii*, *E. campestre*, *E. corniculatum*, *E. glaciale*, and *E. dilatatum* share the same principal compounds in their different fractions, while the root fractions of *E. aquifolium* and *E. maritimum* do not have the same components (Table 1). It would be interesting to analyse the phenology of the essential oils of this species to check whether the season affects its metabolism and the production of terpenoids and their location in the plant. However, the variability of this genus occurs in other species of the same family, where different fractions either share the main compound [87,88] or do not [89,90,91,92,93,94,95,96].

**Table 3:** Essential oil composition (%) of the different parts of *Eryngium dilatatum* Lam. from Spain.

Compound	*I*	I ^1^	E.d.I	E.d.SL	E.d.R
1-(1-methyl-2-cyclopenten-1-yl)-ethanone	885	--	0.6	2.1	t
α-Pinene	932 (1012)	932	2.1	0.5	0.2
Sabinene	963 (1113)	969	t	t	0.1
β-Pinene	970 (1097)	974	t	1.1	0.7
Myrcene	985 (1160)	988	t	--	--
Octanal	993 (1286)	998	3.1	8.1	1.7
*n*-Decane	1000	1000	t	0.2	0.4
α-Phellandrene	1005 (1157)	1002	t	--	--
Benzene acetaldehyde	1009	1036	0.1	0.1	0.4
α-Terpinene	1015 (1173)	1014	t	--	--
*o*-Cymene	1019 (1268)	1022	t	--	--
Limonene	1026 (1191)	1024	2.3	1.9	1.8
β-Phellandrene	1027 (1201)	1025	t	--	--
1,8-Cineole	1029 (1204)	1026	t	--	--
γ-Terpinene	1058 (1240)	1054	t	--	--
Acetophenone	1060	1059	t	t	0.1
Fenchone	1074 (1392)	1083	0.1	0.7	0.2
2-Nonanone	1080	1087	t	--	--
Terpinolene	1085 (1277)	1086	t	--	--
Cryptone	1087 (1669)	1183	0.1	t	0.7
Nonanal	1093	1100	t	--	--
Linalool	1096 (1549)	1095	1.0	1.7	0.2
*cis*-Thujone	1117	1101	t	t	0.3
Menthone	1148 (1468)	1148	t	--	--
Terpinen-4-ol	1175 (1603)	1174	t	--	--
α-Terpineol	1183 (1700)	1186	0.1	--	--
*n*-Dodecane	1200	1200	t	0.6	--
n.i. 1	1235	--	0.1	0.4	--
*trans*-Chrysanthenyl acetate	1241	1235	t	--	--
Carvacrol	1298	1298	0.1	--	--
(*E*,*Z*)-2,4-Decadienal	1301 (1229)	1292	t	--	--
δ-Elemene	1333 (1468)	1335	0.1	0.4	0.1
α-Cubebene	1345 (1455)	1345	0.1	t	0.2
(*Z*)-β-Damascenone	1354	1361	0.1	0.3	t
α-Copaene	1366 (1480)	1374	1.0	1.9	0.6
β-Bourbonene	1376 (1515)	1387	0.7	1.2	0.5
β-Cubebene	1382 (1535)	1387	t	--	--
β-Elemene	1387 (1587)	1389	0.8	2.1	0.4
α-Cedrene	1405 (1669)	1410	t	t	0.2
α-Gurjunene	1406 (1528)	1409	t	t	0.2
β-Cedrene	1409	1419	t	t	0.3
*(E)*-Caryophyllene	1410 (1594)	1417	1.4	2.6	0.6
β-Gurjunene	1426 (1595)	1431	0.2	0.5	0.4
α-*trans*-Bergamotene	1432 (1583)	1432	0.1	--	--
Aromadendrene	1433 (1605)	1439	0.2	0.3	--
α-Guaiene	1434	1437	0.4	0.3	0.9
α-*neo*-Clovene	1445	1452	0.4	--	--
α-Humulene	1447 (1667)	1452	1.6	1.8	0.8
*cis*-Muurola-4(14)-5-diene	1454	1465	t	--	--
*allo*-Aromadendrene	1466	1458	t	--	--
**Germacrene D**	**1476 (1713)**	1484	**46.5**	**38.4**	**9.1**
α-Selinene	1492 (1727)	1498	0.4	0.2	1.4
Bicyclogermacrene	1493 (1750)	1500	3.5	2.5	0.9
α-Muurolene	1499 (1924)	1500	t	--	--
γ-Cadinene	1508 (1691)	1513	0.4	0.7	0.9
*trans*-β-Guaiene	1511 (1611)	1502	t	--	--
β-Bisabolene	1513 (1727)	1505	0.1	t	1.6
Cubebol	1515 (2068)	1514	t	--	--
δ-Cadinene	1522 (1760)	1522	1.9	3.6	2.6
*trans*-Calamenene	1527	1521	0.1	t	0.4
Cadina-1,4-diene	1528 (1783)	1533	0.2	0.5	0.6
α-Cadinene	1539	1537	t	--	--
Elemol	1541 (2085)	1548	t	--	--
β-Calacorene	1553	1564	0.2	t	1.2
n.i. 2	1557	--	0.7	0.4	--
n.i. 3	1559	--	3.3	3.2	4.2
Dodecanoic acid	1568 (2495)	1565	0.3	0.4	0.8
Spathulenol	1576 (2133)	1577	2.5	2.4	4.6
Caryophyllene oxide	1580 (1987)	1582	0.3	--	--
Striatene	1582	--	0.8	0.4	1.5
*cis*-Arteannuic alcohol	1592	1593	0.1	--	--
Carotol	1594 (2026)	1594	0.7	1.3	2.5
β-Oploplenone	1597	1607	t	--	--
n.i. 4	1602	--	1.6	0.7	2.0
n.i. 5	1608	--	0.7	1.0	2.2
β-Bazzanene	1610 (1519)	1519	0.7	1.0	2.0
Cubenol	1633	1645	t	--	--
β-Eudesmol	1638 (2239)	1649	0.4	t	1.4
α-Muurolol	1650 (2246)	1644	2.5	2.1	3.1
α-Cadinol	1651 (2243)	1652	3.8	3.7	4.4
Khusinol	1665	1679	1.8	1.3	3.2
Khusimol	1716	1741	0.4	t	--
14-Oxy-α-Muurolene	1745 (2016)	1767	2.0	0.5	2.6
14-Hydroxy-α-Muurolene	1757 (2026)	1779	0.3	t	0.1
Total			97.3	87.5	76.2

*I* = Kováts retention indices on DB-1 column and on DB-Wax column in parenthesis; I ^1^ = literature linear retention index on DB-5 column [96]; t = traces (% < 0.1); n.i. = not identified; E.d. = *Eryngium dilatatum*; I = inflorescences; SL = stems and leaves; R = roots; n.i. 1 *I* = 1235, 136 [M^+^] (10), 70 (100), 55 (75), 41 (65), 83 (60), 57 (55), 69 (54), 43 (52), 98 (25), 110 (20), 121 (15); n.i. 2 *I* = 1557, 220 [M^+^] (10), 123 (100), 131 (75), 109 (43), 91 (40), 146 (39), 163 (27), 187 (9), 202 (5); n.i. 3 *I* = 1559, 220 [M^+^] (35), 135 (100), 107 (88), 159 (85), 91 (83), 121 (81), 177 (79), 81 (60), 41 (45), 55 (40), 137 (39), 69 (30), 161 (23), 205 (20), 189 (10); n.i. 4 *I* = 1602, 220 [M^+^] (15); 91 (100), 105 (85), 79 (80), 123 (65), 41 (64), 159 (60), 131 (50), 145 (50), 187 (18), 177 (13); n.i. 5 *I* = 1608, 222 [M^+^] (5), 109 (100), 81 (50), 93 (48), 41 (45), 161 (35), 67 (30), 55 (28), 119 (25), 135 (20), 204 (18), 177 (18), 189 (15).

The chemical composition of the different species of *Eryngium* studied to date, are qualitatively and quantitatively different, and they do not share any of the principal compounds (Table 1). Germacrene D is the only compound that appears as the principal component of four of these species (*E. dilatatum*, *E. aquifolium*, *E. campestre*, and *E. maritimum*). The habitat and climatic conditions seem to affect the chemical composition of the species within this genus; therefore, it is expected that species grown under similar ecological conditions would show similar constituents.

Other species of this genus have been reported to have analgesic, anticonvulsant, antifungal, anti-inflammatory, antimicrobial, antimutagenic, antioxidant, antiviral, cytotoxic, haemolytic, inhibitory, larvicidal, molluscicidal, or parasiticidal properties [8,9,10,11,12,13,15,16,17,19,20,25,26,28,29,30,31,39,40,41,45,46,47,49,50,51,52,53,86]. It would be interesting to check any of these properties or activities in the essential oils of this species. In the present paper, the biological properties of the essential oils from the various fractions of this species were not evaluated due to the limited amount of oil available. However, germacrene D, the major component of these oils is a hydrocarbon sesquiterpene for which a number of bioactivities have previously been reported [97,98,99,100]. It would be interesting to study the phenology of this compound in this species to evaluate the usefulness of *E. dilatatum* oils for attracting/repelling insects or for allopathic and antimicrobial activities.

The distribution of the terpenoid compounds in the different fractions of *E. dilatatum* appears at the end of Table 4. All the analysed fractions showed sesquiterpenes as the predominant group, with percentages of 91.6%, 81.6%, and 72.6% for the inflorescences, stems and leaves, and roots, respectively. In the aerial parts, the sesquiterpene hydrocarbons (61.8% and 58.4%) were more abundant than the oxygenated ones (29.8% and 23.2%). In contrast, the roots accumulated a higher amount of oxygenated sesquiterpenes (45.2%) than sesquiterpene hydrocarbons (27.4%). The monoterpene fraction was lower than 5% of the total oil, irrespective of the fraction considered, and was always higher than that of the oxygenated monoterpenes. Most of the *Eryngium* species studied to date that inhabit open and dry areas have sesquiterpenes as the predominant fraction, whereas the species that grow close to lakes or wet soils have high amounts of monoterpenes [72]. Other species of *Eryngium* growing under hard climatic conditions exhibited diterpenes as the main components [73,74]. *E. campestre* seems to modify its chemical composition according to the type of soil [69]. This could also be one of the reasons why none of the compounds described to date for this genus could be used for chemotaxonomy proposes.

## 3. Materials and Methods

### 3.1. Plant Material

Several specimens of *Eryngium dilatatum* were gathered during the summer (15-VII-1999) at flowering in Grazalema, Cádiz (Spain). A voucher specimen (MACB-73213) was lodged at the Herbarium of the Faculty of Biology, Complutense University, Madrid, Spain. More details about the locality and the fractions considered appear in Table 2.

### 3.2. Isolation of Volatile Oils

The different parts of *Eryngium dilatatum*, which included the inflorescences (I), stems and leaves (SL), and roots (R), were air-dried. Each one was cut into small pieces (<3 cm) and subjected, separately, to hydro-distillation with cohobation for 8 h according to the method recommended in the Spanish Pharmacopoeia [101] to obtain their essential oils. In brief, each fraction of the inflorescences (I), stems and leaves (SL), and roots (R) was included in a round-bottom flask. The samples were covered with distilled water, and salt (5 g/L) was added to increase the boiling point. The Clevenger apparatus was tightened to the flask, and it was heated to boiling. After 8 h, the oils were measured (mL) and gathered in topaz vials. All the oils were dried over anhydrous magnesium sulphate and stored at 4 °C in the dark. The yields, based on a dried weight basis, of the different parts analysed appear in Table 2.

### 3.3. Gas Chromatography (GC)

GC analysis was carried out on a Varian 3300 gas chromatograph fitted with a fused silica methyl silicone DB-1 column (50 m × 0.25 mm, 0.25 μm film thickness). The temperature was programmed from 95 °C to 240 °C at 4 °C min^−1^. Injection was performed at 250 °C in the split mode (1:100). Nitrogen was used as the carrier gas (1.5 mL min^−1^). Detection was carried out using a flame ionization detector (FID) at 300 °C. The injection volume for all the samples was 0.1 μL of pure oil.

### 3.4. Gas Chromatography–Mass Spectrometry (GC-MS)

GC-MS analyses were carried out using an Agilent Technologies 6890 gas chromatograph coupled to an HP 5973 mass spectrometry detector. A fused silica SE-30 capillary column (50 m × 0.25 mm, 0.25 μm film thickness) was used for separation. The column temperature was programmed from 70 °C to 220 °C at 4 °C min^−1^, and helium at 1 mL min^−1^ was used as a carrier gas. The mass spectra were recorded in the scan mode at 70 eV.

In order to confirm the identification of several compounds, the oil samples were also analysed using a VG Quattro gas chromatograph–mass spectrometer operating at 70 eV ionization energy. The GC column used was a DB-Wax (60 m × 0.32 mm, × 0.25 μm film thickness) programmed from 35 °C to 220 °C at 3 °C min^−1^ with helium as a carrier gas.

### 3.5. Qualitative and Quantitative Analyses

Most of the constituents were tentatively identified by GC via a comparison of their retention indices with those of the authentic standards available in the authors’ laboratory or with retention indices from the references [102,103,104,105,106,107,108]. Further confirmation of the identifications was achieved by GC-MS; the fragmentation patterns of the experimental mass spectra were compared with those of the mass spectral data of the WILEY and NIST libraries. The other constituents were either synthesised or identified in oils of known composition.

Semiquantitative analysis (percent data) was carried out directly from peak areas in the GC profile.

## 4. Conclusions

This contribution aimed to study the chemical composition of the essential oils of *E. dilatatum* Lam. from the Iberian Peninsula; it represents an advance in the knowledge of this rarely studied species. The different fractions analysed (inflorescences, stems and leaves and roots) yielded low amounts of essential oils. These results seem to be correlated with the environment where this species grows, as other species of this genus that inhabit sandy, dry soils also give rise to low amounts of oil. However, the oil yield of this genus is low in comparison with other tropical species of this genus and other species belonging to the same family that are widely used as culinary herbs.

*E. dilatatum* Lam. showed only one principal compound, Germacrene D (9.1–46.5%), which was present in all the fractions studied. Although a number of bioactive properties have previously been described for this hydrocarbon sesquiterpene, further research should be carried out to confirm its contribution to the bioactivity of *E. dilatatum* essential oils.

With regard to the terpenoid compounds, the essential oils of *E. dilatatum* showed sesquiterpenes as the predominant group in the different fractions analysed: inflorescences, stems and leaves, and roots. As compared to previous studies, it can be concluded that the Iberian Peninsula species of this genus growing under comparable conditions shows similar constituents; so, the ecological conditions seem to affect the chemical composition of this genus to a noticeable extent.

## Figures and Tables

**Table 2:** Oil yield of the different parts of *Eryngium dilatatum* Lam. from Spain.

Sample	Voucher Details	Yield (%)
E.d.I	MACB-73213. Road from Grazalema to Zahara (Km 1), Cádiz province (Spain). All the specimens were gathered during the summer season in flowering period. 15-VII-1999. 30STF7871.	0.29
E.d.SL		0.33
E.d.R		0.14

Yield based on dry weight. E.d. = *Eryngium dilatatum*; I = inflorescences; SL = stems and leaves; R = roots.

**Table 4:** Distribution of terpenoid compounds in the different fractions of *Eryngium dilatatum* Lam. from Spain.

Terpenoid/Sample	E.d.I	E.d.SL	E.d.R
Monoterpene hydrocarbons	4.4	3.5	3.1
Oxygenated monoterpenes	1.3	2.4	0.5
Sesquiterpene hydrocarbons	61.8	58.4	27.4
Oxygenated sesquiterpenes	29.8	23.2	45.2
Total	97.3	87.5	76.2

E.d. = *Eryngium dilatatum*; I = inflorescences; SL = stems and leaves; R = roots.

## Data Availability

Data are contained within the article.
